# Reflections Suggested by the Perusal of the Lectures of Sir Astley Cooper

**Published:** 1847-07

**Authors:** C. Colegrove

**Affiliations:** Sardinia


ART. IV.—*Reflections suggested by the perusal of the lectures of Sir Astley Cooper.* By C. COLEGROVE.

DR. FLINT:—You publish cases—I wonder if you are favorably inclined toward speculations, comparisons, deductions, inquiries, &c., if they are reasonable and scientific, albeit there is not a pictured sprinkling of pains, diagnosis, terminations, and the varieties of medical record! Now I am not a venerable, chin-furrowed practitioner,—my notes of cases *are* brief and innumerous; but, *sum studens*, i. e., *qui literarum studio operam dat.* And *sed epistola est tam polita, quæ, nisi a studente, non potest scribi*, says Pliny. Pray, pardon my egotism. Forsitan, you will say I belie my quotation, or it condemns me. I am determined to try my fortune and pen, however, and if, (I know it is perilous) my undertaking makes me unpopular, and is a *contributary* bore, *from me*, *editorial* bore, *to thee*, toss my presumptuous furnishing into the stove, and I shall doubtless, be quill-dumb, a six-month. But if you scrupulously can, deign indulgence to my vanity and lack-ceremony. I always need—always expect clemency and consideration, otherwise I am a helpless, hopeless, forlorn superfluent, and a *dependent* I am compelled to be,—so is every body else.

My Dear Sir:—I have just finished the second reading of Cooper's Lectures. Dr. Rush, venerable, distinguished, erudite man!—in one of his Introductry Lectures, has this paragraph:—"Let us finally remember that there is a great difference between reading and study. The former may be compared to exercise, the latter to bodily labor. We read history and travels, but we study books of science. We rise refreshed from reading Robertson's history of Charles V. and Bruce's search for the origin of the Nile, but we seek for repose, after studying the works of Sydenham and Hartly."

Sir Astley Cooper's Lectures on Surgery, in some respects imperfect, (and unequal to the original, as published, they doubtless are,) appear to me altogether beyond praise. I do not hesitate to own, the work involuntarily excited my highest, most disinterested admiration, not only of itself, its merit, its brief, perspicuous, logical style, its peculiar independence of language and reasoning, with its multitude of adduced examples, but, in natural course, of the renowned author also. Whether to place this in the first or last description of books, given by Dr. Rush, I hardly know. In fact, it belongs to both. It is study and relaxation at once, to peruse this volume. In the world of medical books, some of which I have seen, far fewer read, I am candidly unable to indicate one on Surgery, that in my judgment, is *so* comprehensible, clear, agreeable, dignified, improlix, and valuable to the student. Before my eyes, are many volumes. I read the names of Gregory, Good, Bell, Eberle, Thomas, and so forth. Now I had a neighbor—a year or two since, who occasionally inclined to peer into medical transactions, and written practice, and he had some discrimination. He could never, patiently, find the bottom of his page. He twisted, hemmed, spat, looked sometimes indignant, or scornful, yet persevered a little longer; and then in abrupt disregard of decorum, and defiance of all my desperately struggled for contradictory influence, gave the book a terrible blow with his clenched fist, or a contemptuous toss to a remote corner of the office, vehemently declaiming against the interminable and irrelevant references, theories, opinions, and all this like, that abound so intolerably in works on medicine. Mr. editor, he was half right, was he not? Is it not a characteristic dishonor to the medical art, and literature, that so many, and so speculative, silly, tedious, labyrinthine controversies, fancies, and theories, should find access to the best specimens of medical authorship? And how, or when, will this defect be remedied? Surely, no sensible reader can deny the great superiority of the work in question, on this very account. I write, unpredisposed. Limit, point, concise description, and an absolute rejection of theories, and whatever could be fancied superfluous, these qualities, so foreign to medical publications, *en masse*, render Sir A. Cooper's work and fame, doubly dear, and intrinsically superior. And when the requisites for professional permit, professorial adoption, and the common dignity of M. D., are so inconsiderable and a-stringent, is it not of the highest consequence that elementary books should be comprehensive, concise, straightforward in style, and void of adductions that complicate and discourage the inquisitive mind? Here is an expansive theme. How can I do it justice? Bear with me a moment longer. Who are the devoters of medical science? Many are young men of early and thorough education, faithful, laborious, and persevering discipline, and strong intelligence; but *the more*, rise, like an army from sowen dragon, teeth, unlettered, from the plough-furrow, and march straight into the dissecting room, in shirt sleeves, filling out according to statute, alternately in the physician's office, and the department of lectural instruction, the mere hasty three years of apprenticeship, study, and initiatory practice. It is true, many of this class, through great native energy and endowments, with constant practice and study, ultimately attain great eminence, and become the brightest ornaments of the profession. But how can it be posible that the period of preparation so brief, should be at all adequate, especially, since authors are so voluminous, theoretical, and contentious? That to investigate, treat, and control diseases, receive the responsibility of life or death continually, and plunge the surgical knife into an organization the most extensive, compound, and wonderful, should need less than half the patience and toil which are indispensable to the adjudication of contests, pecuniary interests, or even to the exercise of judicial functions! If these desultory suggestions are not unacceptable, I shall be most happy, at another time, to prolong the examination of a highly interesting and vitally important subject. To the genius, skill, decision, wisdom, mental strength, and acquirements—to the universal fame of Sir Astley Cooper, I can pay no meet tribute. Lest I offend, by prolonging this article too far, I close—saying, I am

Truly, and respectfully Yours,

C. Colegrove.

Sardinia, June 5th, 1847.

